# Yangyin Qingre Huoxue Method in Traditional Chinese Medicine Ameliorates Atherosclerosis in ApoE^−/−^ Mice Suffering from High-Fat Diet and HSP65 Aggression

**DOI:** 10.1155/2019/2531979

**Published:** 2019-01-01

**Authors:** Runze Qiu, Jun Long, Liyu Zhou, Yuanjing Ma, Lingang Zhao, Fumin Liu, Dongping Yuan

**Affiliations:** ^1^School of Pharmacy, Jiangsu Key Laboratory for Pharmacology and Safety Evaluation of Chinese Materia Medica, Nanjing University of Chinese Medicine, Xianlin Dadao 138, Nanjing, 210023, China; ^2^Jiangsu Province Hospital of TCM, Affiliated Hospital of Nanjing University of Traditional Chinese Medicine, Hanzhong Road 155, Nanjing, 210029, China

## Abstract

Atherosclerosis (AS) is a complicated arterial disease resulting from abnormal lipid deposition and inflammatory injury, which is attributed to Yin deficiency, accumulation of heat materials, and stasis of blood flow in Traditional Chinese Medicine (TCM) theory. Thus, according to TCM theory, the method of nourishing Yin (Yangyin), clearing away heat (Qingre), and promoting blood circulation (Huoxue) is a reasonable strategy, which has achieved remarkable clinical efficacy in the treatment of AS, but the mechanisms remain to be known. In this study, we evaluated the effects of Yangyin Qingre Huoxue Prescription (YQHP) on AS in ApoE^−/−^ mice suffering from a high-fat diet and heat shock protein (HSP65) attack. YQHP regulated levels of blood lipids and inflammation-linked cytokines as well as Th17/Treg ratio in peripheral blood. Suppressed IL-6-p-STAT3 signaling and restored IL-2-p-STAT5 signaling in the presence of YQHP may partake in the regulation of Th17 and Treg differentiation. Moreover, YQHP modulated transcriptional levels of costimulator CD80 in aortas as well corresponding to the downregulation of GM-CSF in serum and CD3 expression in CD4^+^ T cells, which might indicate the potential of YQHP to regulate antigen presenting cells. All these effects eventually promoted the improvement of atherosclerotic lesions. In addition, YQHP promoted less monocyte infiltration in the liver and lower levels of AST, ALT, and AKP production than simvastatin. Conclusively, lipid-regulating and anti-inflammatory functions mediated by YQHP with lower hepatotoxicity than simvastatin hindered the progression of HSP65 aggravated AS in ApoE^−/−^ mice, indicating the effectiveness of Yangyin Qingre Huoxue Method in the treatment of AS.

## 1. Introduction

Atherosclerosis (AS), a chronic inflammatory disease featuring lipid-deposited vascular wall with rupture-prone plaque [1, 2], is an important pathogenesis of cardiovascular and cerebrovascular accidents [3]. Numerous studies have shown that immune disorder is a crucial cause of AS [4], and the accumulation of inflammatory factors in the context of immune disorder contributes to the formation of vascular lesions [5], which can lead to subsequent thrombosis and poor blood flow and eventually induce and aggravate AS with excess lipid burden.

According to Traditional Chinese Medicine (TCM) theory, blood stasis and poor circulation in the AS milieu are attributed to the accumulation of heat and toxic factors [6], while Yin deficiency, which has a great connection with immune disorder [7], is the basic condition for the accumulation of such factors [8]. Therefore, the method of Yin nourishing (Yangyin), removal of heat and toxic materials (Qingre), and invigoration of blood circulation (Huoxue) originated from TCM theory provides the possibility of comprehensive and effective treatment of AS. Prepared* Reynoutria multiflora* (Thunb.) Moldenke,* Polygonatum sibiricum* F. Delaroche with Yangyin effect,* Reynoutria japonica* Houtt and* Stemmacantha uniflora* (L.) Dittrich with Qingre effect, and* Curcuma longa* L. and* Carthamus tinctorius* L. with Huoxue effect (Chinese medicine details are listed in Table 1) in the recipe of Yangyin Qingre Huoxue Prescription (YQHP) were combined to treat dyslipidemia and immune disorders in the atherosclerotic context [8–11]. The herbs in YQHP have shown exact lipid-regulatory, anti-inflammatory, and blood circulation ameliorative functions in clinical or animal studies (Table 1). Moreover, as an in-hospital preparation (i.e., Luhuang granule) in Jiangsu Province Hospital of TCM in China, YQHP has been traditionally used in cardiology for many years and displayed significant efficacy in nearly 100 cases of hyperlipidemia and atherosclerotic patients with Yin deficiency [12–14]. It reduced plaque area and intima-media thickness, controlled blood lipids and inflammatory factors, and improved blood rheology indicators without serious adverse reactions in clinical and animal studies [8, 15, 16]. However, the anti-inflammatory mechanisms of YQHP for the treatment of AS have not been investigated in depth.

It is known that the status of T cell activation and differentiation determines the progression of inflammation in AS [30, 31]. When major histocompatibility complex class II (MHC-II) in antigen presenting cells (APCs) such as dendritic cells (DCs) activated T cell receptor (TCR) on the cell membrane, signals transmitted to CD3 chains induce T cell activation [32, 33], which is the first signal. Physiological T cell responses also require costimulation crosstalk, the second signal involving CD80/CD86 on APCs and CD28 on T cells [34]. Cytokines released from DCs, as the third signal, differentiate CD4^+^ T cell into various subtypes, among which regulatory T cells (Tregs) and helper T cells 17 (Th17s) play the most prominent roles in AS [35, 36]. Treg cells expressing the forkhead box P3 (Foxp3) and CD25 are promoted from IL-2-p-STAT5 pathway [37, 38] and show immunosuppressive functions, which can inhibit the activation and differentiation of T cells [39–41] and production of inflammatory factors such as IFN-*γ*, TNF-*α*, and MCP-1 in macrophages and Th cells by secreting TGF-*β*, IL-10, and other inhibitory cytokines [42, 43]. Correspondingly, the mighty inflammatory Th17 cells expressing nuclear receptor ROR-gamma (ROR*γ*t) are induced by IL-6-p-STAT3 channel, which excrete IL-17 and IL-22 to mediate inflammation formation and progression [44, 45].

Heat shock protein 65 (HSP65), which is highly homologous with HSP60 and an autoantigen detected in human and animals [46], could trigger the reaction to autoreactive T cells [47] and endothelial stress [48] to exacerbate the inflammatory response and plaques in AS. A vaccine against HSP65 originated from* Mycobacterium tuberculosis* (mHSP65) improved AS through nasal immunization with controlled levels of serum lipids and cytokines during our early studies on rabbits [49, 50]. As shown in the results, mHSP65 aggravated the lesions and inflammation of AS in apolipoprotein E knockout (ApoE^−/−^) mice with the elevated Th17/Treg ratio, testifying proinflammatory and atherogenic effects of mHSP65, which reflected the complex atherogenic background caused by both high-fat diet and bacterial infections.

YQHP immensely improved the disorder of total cholesterol (TC), triacylglycerol (TG), low density lipoprotein cholesterol (LDL-C), and high density lipoprotein cholesterol (HDL-C) in serum and the evolvement of plaque in the aorta in the backgrounds of the western diet and HSP65 boost. During YQHP treatment, reduced ratio of Th17/Treg cells with suppressed IL-6-p-STAT3 and restored IL-2-p-STAT5 signaling were confirmed, indicating the regulatory functions upon the differentiation of the two types of cells. Reduced levels of costimulatory molecules such as CD80 and CD86* in vivo* and blocked T cell activation suggested the probable effects focus on APCs. These results illustrated the lipid-regulating and anti-inflammatory potential of YQHP in the treatment of AS caused by high-fat diet and HSP65.

## 2. Materials and Methods

### 2.1. Animal Procedure

ApoE^−/−^ mice [42-55 days, 18-22 g, male, specific pathogen free grade, certificate number: 11400700229437, permit number: SCXK (JING) 2016–0011] were purchased from Beijing Vital River Laboratory Animal Technology Co., Ltd. on a C57BL/6J background and raised in Nanjing University of Chinese Medicine Experimental Animal Center (Specific Pathogen Free grade). The laboratory animal protocols were approved by Nanjing University of Chinese Medicine Experimental Animal Center and conducted in accordance with the “Principles for Use of Animals” and “Guide for the Care and Use of Laboratory Animals” of the U.S. National Institutes of Health. Recombinant HSP65 proteins originating from the* Mycobacterium tuberculosis* were purified according to the reported method [50]. After one week of accommodation to the environment, mice were weighted and fasted overnight and serum samples collected from the orbital cavity. As shown in Table 2, apart from the control group, mice in other groups were fed with a western diet (1.25% cholesterol and 20% lard) for 20 weeks. Mice in western diet plus HSP65, simvastatin, and YQHP groups were subcutaneously injected with HSP65 (0.01mg dissolved in sterilized PBS) additionally at weeks 1, 4, and 7. The intragastric administration of simvastatin or YQHP was synchronized with the western diet (simvastatin: 3.3 mg/kg, Merck Sharp & Dohme Ltd, dissolved in distilled water; YQHP: 18 g/kg), while mice in other groups were given the equal volume of normal saline. At the 10th and 20th week, serum samples were collected from the orbital cavity for the second and third times with overnight fast. At the 21st week, mice were weighed, fasted overnight, anesthetized using isoflurane, and euthanized. Fresh anticoagulant blood samples were collected from the left ventricle for flow cytometry analysis. Aortas, hearts, spleens, livers, and thymuses were separated and frozen in -80 degrees or soaked in 4% paraformaldehyde (PFA).

### 2.2. Prescription Preparation and Quality Inspection

The subterranean parts of Prepared* Reynoutria multiflora* (Sichuan, China),* Polygonatum sibiricum* (Yunnan, China)*, Reynoutria japonica* (Jiangxi, China),* Stemmacantha uniflora* (Hebei, China), and* Curcuma longa* (Sichuan, China) and aerial parts of* Carthamus tinctorius* (Sinkiang, China) have been identified by Dr. Shengjin Liu (School of Pharmacy, Nanjing University of Chinese Medicine). A voucher specimen (No. nzy-yl-170715) including the six medicinal herbs was deposited at the Chinese Medicine Herbarium of Nanjing University of Chinese Medicine.

The dried subterranean parts of five kinds of medicinal herbs were powdered and mixed with the aerial parts of* Carthamus tinctorius*. The mass ratio of* Reynoutria multiflora*,* Polygonatum sibiricum, Reynoutria japonica*,* Stemmacantha uniflora*,* Curcuma longa*, and* Carthamus tinctorius* in YQHP was 1: 1: 3: 3: 1: 1. The mixed powder was soaked in 50% ethanol for 12 h at room temperature and extracted 3 times via reflux method. The extracted solution was filtrated, concentrated to a semisolid mass, dried, and dissolved to 1.8g/ml with distilled water. The high performance liquid chromatography (HPLC) method was used to identify YQHP extract dissolved with methanol, and the operation strictly followed the Pharmacopoeia of the People's Republic of China [9]. As shown in Supplementary Fig.  S1 online, positive peaks of main ingredients including 2,3,5,4′-tetrahydroxyl diphenylethylene-2-o-glucoside (diphenyl diglycoside, Fig.  S1A, the quality inspection standard of* Reynoutria multiflora* detected at 320 nm, mobile phase: 17% acetonitrile and 83% water), polydatin (Fig.  S1B, the quality inspection standard of* Reynoutria japonica* detected at 300 nm, mobile phase: 40% acetonitrile and 0.2% phosphoric acid solution), curcumin (Fig.  S1C, the quality inspection standard of* Curcuma longa* detected at 425 nm, mobile phase: 48% acetonitrile and 0.2% glacial acetic acid solution), hydroxysafflor yellow A (Fig.  S1D, the quality inspection standard of* Carthamus tinctorius* detected at 403 nm, mobile phase: 26% methanol, 2% acetonitrile and 0.2% phosphoric acid solution), and *β*-ecdysone (Fig.  S1E, the quality inspection standard of* Stemmacantha uniflora* detected at 247 nm, mobile phase: 17% acetonitrile and 83% water) appeared smoothly. The content of each inspection component in YQHP has been shown in Supplementary Table S1 online.

### 2.3. Serum Lipid Analysis

Enzymatic biochemical kits (Nanjing Jiancheng) were used to determine serum lipids via microplate reader (TECAN): Total Cholesterol Assay kit (Cat A111-1), Triacylglycerol Assay kit (Cat A110-1), Low Density Lipoprotein Cholesterol Assay kit (Cat A113-1), and High Density Lipoprotein Cholesterol (Cat A112-1).

### 2.4. Atherosclerotic Lesion Staining and Analysis

The aortas from the aortic arch to left and right common iliac artery were separated and excess tissues outside the vessels were carefully removed. Then the aortas were fixed in 4% PFA and stained with the oil red O. Before photograph, aortas were rinsed in 75% ethanol to differentiate normal tissue into creamy white and washed. The areas of aortic plaque were measured by MapInfo 7.0 (USA, https://www.mapinfo.com). The hearts were fixed with PFA, embedded in paraffin, sliced latitudinally, stained by hematoxylin-eosin (H&E), and shot by a digital microscope (magnification: × 400).

### 2.5. Hepatotoxicity Analysis

ApoE^−/−^ mice from the same source were separate from other trials. Mice were divided into 4 groups including control, western diet, simvastatin, and YQHP group. Besides the control group, mice were fed with the western diet. The dose of simvastatin was 1.1mg/kg, which is the minimum clinical equivalent dosage within the therapeutic window, while YQHP was still on the dosage of 18 g/kg. Western diet and drug administration lasted for 30 days. At the 31st day, mice were weighted, fasted overnight, and euthanized. Livers were fixed by PFA, stained with H&E, and evaluated by a professional pathologist. Serum samples were collected from the left ventricle. The vitality of aspartate aminotransferase (Cat C010-2), alanine aminotransferase (Cat C009-2), and alkaline phosphatase (Cat A059-2) was measured by microplate kits (Nanjing Jiancheng Ltd.) and calculated by Carmen or King units.

### 2.6. ELISA Assay

The levels of IL-2, IL-4, IL-6, IL-10, IL-17, IL-22, TGF-*β*, and GM-CSF in serum were determined by ELISA kits (Nanjing Maibo): Mouse IL-6 ELISA kit (Cat MBE10288), Mouse IL-17 ELISA kit (Cat MBE10413), Mouse IL-22 ELISA kit (Cat MBE10304), Mouse IL-2 ELISA kit (Cat MBE10294), Mouse TGF-*β* ELISA kit (Cat MBE10097), Mouse IL-10 ELISA kit (Cat MBE10477), Mouse GM-CSF ELISA kit (Cat MBE10484), and Mouse IL-4 ELISA kit (Cat MBE10290). All operations followed the instructions. After centrifugation, the serum samples were diluted 5 times and then were applied to each well on the plate. The standards or normal saline were added to the standard wells or blank wells, respectively. Then horseradish peroxidase (HRP)-labeled detection antibody was added to each well and incubated at 37°C for 60 min. After the incubation, the liquid was discarded and the plate was washed 5 times and patted dry. The substrates A and B were added to each well and incubated at 37°C for 15 min in the dark. Finally, the stop solution was added to each well, and the optical density (OD) value of each well was measured at a wavelength of 450 nm within 15 min. Standard curves were drawn based on each standard concentration and OD value. The cytokine concentration of the sample is calculated according to the standard curve and the OD value of the sample well (the OD value of the blank well is subtracted per well). The r^2^ value of each standard curve is greater than 0.995.

### 2.7. Flow Cytometry

Fresh blood samples were anticoagulated with heparin at a final concentration of 1 mg/ml. Subsequent sample processing and flow cytometry detection were commissioned by KeyGEN BioTECH (China). Samples were centrifugated by lymphocyte separation solution (Yuyang Biological) to obtain pure lymphocytes and then fixed with the Fixation Buffer (BD Cytofix). Before measurement, samples were washed, treated with the Fixation/Permeabilization Solution (BD Pharmingen) and Phorbol ester (Sigma, for Th17), incubated with flow cytometry antibodies (BD Pharmingen), and resuspended in phosphate buffered saline (PBS). Then the samples were determined with BD FACSCalibur Flow Cytometer. Antibody collocations were as follows:

- CD3^+^CD4^+^ T cells: FITC Rat Anti-Mouse CD4 (Cat 553046) and PE Rat Anti-Mouse CD3 (Cat 561799)
- IL-17A^+^CD4^+^ Th17 cells: FITC Rat Anti-Mouse CD4 (Cat 553046) and Alexa Fluor 647 Rat Anti-Mouse IL-17A (Cat 560224)
- CD25^+^Foxp3^+^CD4^+^ Treg cells: FITC Rat Anti-Mouse CD4 (Cat 553046), APC Rat Anti-Mouse CD25 (Cat 561049) and PE Rat Anti-Mouse Foxp3 (Cat 563101).

### 2.8. Quantitative Real-Time PCR

Total RNA was extracted from spleens or aortas by the TRIzol Reagent (Ambion). 100 ng of spleen RNA and 40 ng aorta RNA were quantified with NanoDrop One (Thermo Scientific) and reverse transcribed using 5X All-In-One RT MasterMix (abm) preformed in Veriti 96-Well Thermal Cycler (Applied Biosystems). Quantitative real-time PCR was performed in 7500 Real-Time PCR (Applied Biosystems) using specific primers (synthesized by Sangon Biotech) and EvaGreen 2X qPCR MasterMix-Low ROX (abm). Reverse transcription and amplification conditions followed the reagent instructions. Data were analyzed via the 2^-ΔΔCt^ method normalized to Gapdh. Sequences of primers were used as follows from 5′ to 3′ extremity:

- Gapdh: AGGTCGGTGTGAACGGATTTG (F); TGTAGACCATGTAGTTGAGGTCA (R)
- Stat3: CAATACCATTGACCTGCCGAT (F); GAGCGACTCAAACTGCCCT (R)
- Stat5a: CGCCAGATGCAAGTGTTGTAT (F); TCCTGGGGATTATCCAAGTCAAT (R)
- Jak2: TTGTGGTATTACGCCTGTGTATC (F); ATGCCTGGTTGACTCGTCTAT (R)
- Socs3: ATGGTCACCCACAGCAAGTTT (F); TCCAGTAGAATCCGCTCTCCT (R)
- Foxp3: CACCTATGCCACCCTTATCCG (F); CATGCGAGTAAACCAATGGTAGA (R)
- Tgfb1: TGACGTCACTGGAGTTGTACGG (F); GGTTCATGTCATGGATGGTGC (R)
- Cd80: ACCCCCAACATAACTGAGTCT (F); TTCCAACCAAGAGAAGCGAGG (R)
- Cd86: AGTGATCGCCAACTTCAGTGAACC (F); GGTGACCTTGCTTAGACGTGCAG (R).

### 2.9. Western Blot

Total proteins in spleens or aortas were extracted via High Efficiency RIPA Lysis Buffer (KeyGEN BioTECH), quantified by BCA protein concentration assay kit (Beyotime Biotechnology), and denatured in boiling water with SDS-PAGE protein loading buffer (Beyotime Biotechnology). 30 *μ*g of proteins per sample was loaded in 10% SDS-PAGE gel and transferred onto polyvinylidene fluoride membranes (Merck Millipore). Skim milk in TBST (TBS containing 0.1% Tween 20) was used as blocking agent before antibody incubation. Polyvinylidene fluoride (PVDF) membranes were incubated with rabbit antibodies against p-STAT3-Y705 (Abcam), p-STAT5-Y694 (Cell Signaling Technology), SOCS3 (Abcam), FOXP3 (Abcam), *β*-ACTIN (Abways), and Goat anti-rabbit IgG with HRP (Biosharp), respectively. The imaging was performed in Gel Doc XR Biorad (Bio-Rad) using Chemiluminescent HRP Substrate (Merck Millipore). Blot data were analyzed with Image Lab 4.0 (Bio-Rad). Gray values of blot areas are measured, and the relative expression amount of the protein samples is calculated by the method of the target protein gray value/internal reference *β*-ACTIN gray value.

### 2.10. Statistics

Data are described as mean ± SEM and P<0.05 is considered statistically significant. One-way ANOVA was used for statistical analysis via GraphPad Prism 5.02 software.

## 3. Results

### 3.1. The Lipid-Regulating Capacity of YQHP in Different Observation Period

**Figure 1:**
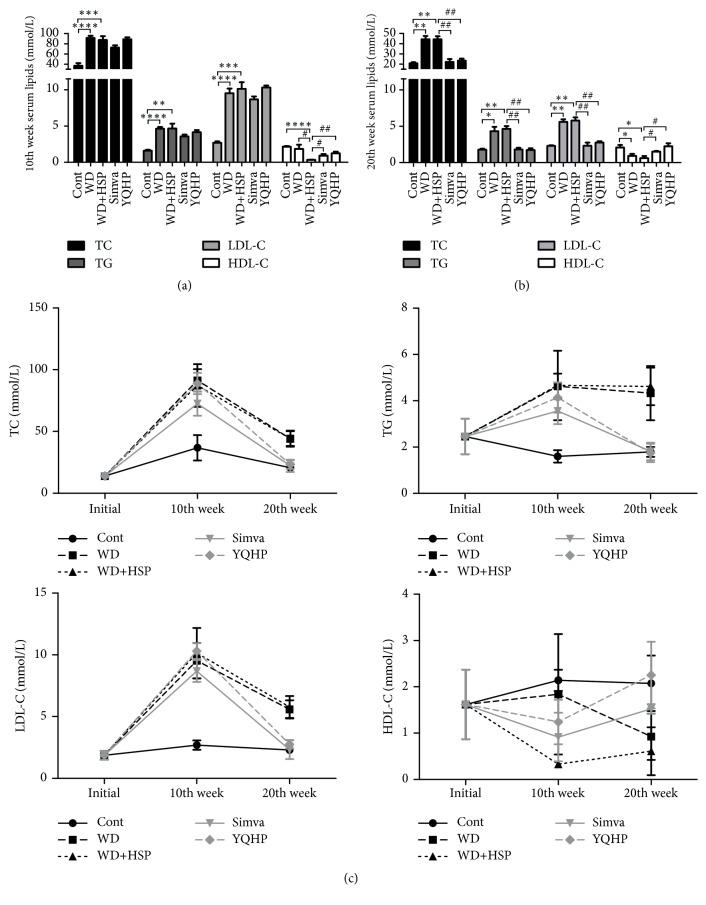
*Effects of YQHP on serum lipids.* (a) TC, TG, LDL-C, and HDL-C from the serum of ApoE^−/−^ mice measured by enzyme method assessing kit at 10th week. (b) TC, TG, LDL-C, and HDL-C from the serum of ApoE^−/−^ mice measured at 20th week. (c) Changes of TC, TG, LDL-C, and HDL-C levels of ApoE^−/−^ mice before modeling, at 10th and at 20th week. The bars or lines shown in the figures represent control (Cont), western diet (WD), western diet plus HSP65 (WD+HSP), simvastatin (Simva), or YQHP group, which are the same with figures below. Bars (a, b) and plots (c) are expressed as mean; error bars: SEM; n value: 6; ^*∗*^P<0.05, ^*∗∗*^P<0.01, ^*∗∗∗*^P<0.001, ^*∗∗∗∗*^P<0.0001 versus the control group; ^#^P<0.05, ^##^P<0.01 versus the WD+HSP group.

Considering that AS is induced by hyperlipidemia, we first tested the levels of serum lipids in ApoE^−/−^ mice. Compared to a normal diet, western diet elevated TC, TG, and LDL-C dramatically at the 10th week, but no further variety of serum lipids were observed with HSP65 injection or YQHP treatment except HDL-C (Figure 1(a)). However, at the 20th week, YQHP downgraded the levels of TC, TG, and LDL-C and restored the concentration of HDL-C similar to simvastatin (Figure 1(b)), the lipid-lowering medicine.

It is noteworthy that the levels of TC and LDL-C varied in two stages during the western diet, but TG was not decreased without drug intervention (Figure 1(c)). Up to 20 weeks of western diet decreased the level of HDL-C, while further injection of HSP65 kept it at a low level since 10th week, indicating that HSP65 has an accelerated effect on dyslipidemia. Besides, both simvastatin and YQHP treatment for 20 weeks showed better controlling functions on dyslipidemia rather than that for 10 weeks (Figure 1(c)), pointing out the importance of long-term treatment in hyperlipidemia.

### 3.2. YQHP Impedes the Progress of Atherosclerotic Lesions

**Figure 2:**
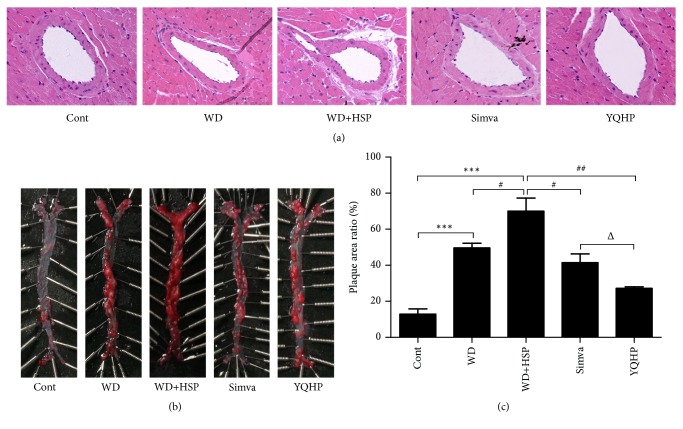
*Effects of YQHP on atherosclerotic lesions.* (a) The photos of the left coronary arteries freshly separated from ApoE^−/−^ mice and stained by H&E after 20 weeks (magnification: × 400, scale bar: 50 *μ*m). (b) The photos of aortic intima of ApoE^−/−^ mice stained by Oil Red O. The aortas were separated from aortic arch to left and right common iliac artery. (c) The areas of aortic plaque measured by MapInfo 7.0. Bars are expressed as mean; error bars: SEM; n value: 4; ^*∗∗∗∗*^P<0.001 versus the control group; ^#^P<0.05, ^###^P<0.005 versus the WD+HSP group; ^Δ^P<0.05 versus the Simva group.

As shown in Figure 2(a), serious stenosis and deformation in hyperlipidemia mice were observed in the coronary arteries stained with H&E after 20 weeks, which were further aggravated with HSP65 injection. Nevertheless, the lumina of coronary arteries in YQHP or simvastatin treated mice was broadened a lot, suggesting potential improvement effect of drug treatment on the blood supply of coronary arteries.

Apart from the coronary artery, another artery with a high incidence of AS is the aorta, particularly the area of the aortic arch. The plaques from the aorta lesion area are a risk factor for rupture and thrombosis as well as considerable inflammation [51], which bring about the poor prognosis of the disease. As shown in results, mice fed with high-fat diet were subject to serious lesions in aortic intima, which further deteriorated to be almost completely covered by plaques after the HSP65 assault (Figures 2(b) and 2(c)), indicating that HSP65 can further aggravate AS on the basic of hyperlipidemia. Encouragingly, the pathological changes of aortas were repressed by YQHP, and the ratio of the nonplaque region in YQHP-treated mice was even higher than that in mice with simvastatin disposal (Figures 2(b) and 2(c)).

During our trial, more than one-third of mice under simvastatin treatment died, which may be relevant to the hepatotoxicity of simvastatin [52, 53]. Therefore, we further investigated the effects of YQHP on livers with a common dose, by comparing to the effects of the minimum clinical equivalent dosage of simvastatin on the liver. As a result, under the background of the western diet, the structure of hepatic lobules was destroyed, and the portal area was wrapped in pseudolobules with mononuclear cell infiltration (see Supplementary Fig.  S2A online). Although hepatic lobules in western diet fed mice treated with simvastatin were almost intact, monocyte infiltration in the portal area was remarkably aggravated (Fig.  S2A), indicating severe liver injury. Correspondingly, during YQHP treatment, both controlled monocyte infiltration and repaired hepatic lobular structure were confirmed (Fig.  S2A). Furthermore, simvastatin treated mice displayed doubled levels of aspartate aminotransferase (AST), alanine aminotransferase (ALT), and alkaline phosphatase (AKP), which reflect disordered liver function [54] (Fig.  S2B-D). In contrast, there was no change under YQHP treatment, and the levels of ALT and AKP were evidently lower than simvastatin group (Fig.  S2C, D). Data from above prompt better efficacy and lower hepatotoxicity of YQHP in AS intervention compared to simvastatin.

### 3.3. The Regulatory Effects of YQHP on Serum Cytokines

**Figure 3:**
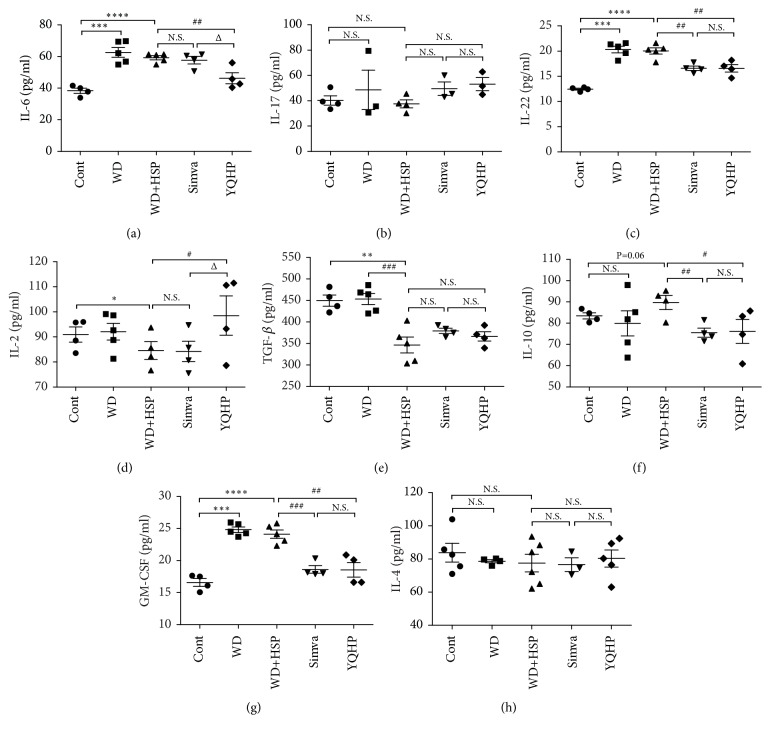
*Effects of YQHP on inflammation-related cytokines in the serum.* (a-h) The levels of IL-6, IL-17, IL-22, IL-2, TGF-*β*, IL-10, GM-CSF, and IL-4 in the serum of ApoE^−/−^ mice collected at 20th week and gauged with enzyme linked immunosorbent assay (ELISA) kits. Bars are expressed as mean; error bars: SEM; ^*∗*^P<0.05, ^*∗∗*^P<0.01, ^*∗∗∗*^P<0.001, ^*∗∗∗∗*^P<0.0001 versus the control group; ^#^P<0.05, ^##^P<0.01, ^###^P<0.001 versus the WD+HSP group; ^Δ^P<0.05 versus the Simva group; N.S.: not significant.

Owing to the aggravated lesion under the offensive of HSP65 (Figures 2(b) and 2(c)), we next investigated the effects of YQHP on inflammation-related cytokines at the 20th week. IL-6, which is the inducer of Th17 cells [44], experienced a huge surge in concentration after mice fed with the western diet. However, the concentration of IL-6 was reduced by YQHP (Figure 3(a)), not simvastatin, a lipid-lowering medicine with strong anti-inflammatory activity in AS [55, 56]. YQHP could just restrain the production of IL-22 raised by high-fat diet with HSP65 treatment, but no change was confirmed in cytokine IL-17 secreted by Th17 in YQHP or simvastatin group (Figures 3(b) and 3(c)).

In contrast, reduced secretion of Treg-inducing cytokine IL-2 was detected in hyperlipidemia mice injected with HSP65, which can be restored by YQHP rather than simvastatin (Figure 3(d)). In addition, HSP65 remarkably downregulated the production of TGF-*β*, which is an inhibitory cytokine secreted from Treg and recuperated faintly by YQHP (Figure 3(e)). But another characteristic immunosuppressive cytokine IL-10 reduced at the disposal of simvastatin or YQHP (Figure 3(f)). GM-CSF, which is the exciter of hematopoietic precursor cells and can induce the generation of APCs including DCs [57], could also be produced by Th17 cells [58] and sustained inflammation status. YQHP and simvastatin drastically decreased the level of GM-CSF lifted by western diet, not HSP65 (Figure 3(g)). But neither modeling nor drug intervention could influence IL-4 (Figure 3(h)), another inducing factor of DC, in our trial. It can be generalized that YQHP controlled the inflammation-related factors in the serum of AS mice principally through the regulation of cytokines stimulating the differentiation of Th17 or Treg cells.

### 3.4. YQHP Regulates Th17/Treg Balance and T Cell Activation in Peripheral Blood

**Figure 4:**
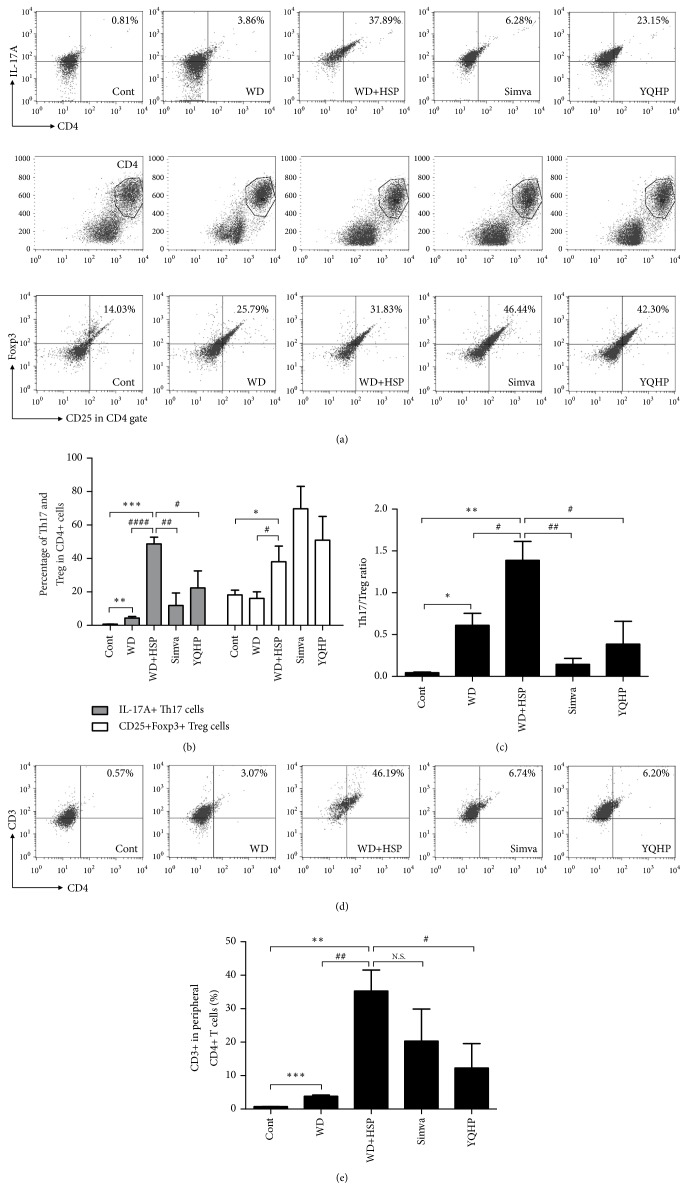
*Effects of YQHP on the levels of CD3* ^*+*^ *CD4* ^*+*^ *T cell, Th17, and Treg in the peripheral blood.* (a, b) The percentage of IL-17A^+^CD4^+^ Th17 and CD25^+^Foxp3^+^CD4^+^ Treg cells determined by flow cytometry. (c) The ratio of IL-17A^+^CD4^+^ Th17/CD25^+^Foxp3^+^CD4^+^ Treg cells. (d, e) The percentage of CD3^+^CD4^+^ TCR-activated T cells determined by flow cytometry. Fresh blood samples from ApoE^−/−^ mice were extracted by lymphocyte separation solution before antibody staining after 20 weeks of modeling and administration of medicines. Bars are expressed as mean; error bars: SEM; n value: 5; ^*∗*^P<0.05, ^*∗∗*^P<0.01, ^*∗∗∗*^P<0.001, ^*∗∗∗∗*^P<0.0001 versus the control group; ^#^P<0.05, ^##^P<0.01, ^####^P<0.0001 versus the WD+HSP group; N.S.: not significant.

On the basis of the modulated levels of IL-6 and IL-2 (Figures 3(a) and 3(d)), we hypothesized that YQHP has an effect on the differentiation of Th17 or Treg cells in AS. To test it, we collected peripheral blood after 20 weeks, and confirmed elevated levels of IL-17A^+^CD4^+^ Th17 cells from mice taking western diet were terribly further increased by a large margin under HSP65 attack (Figures 4(a) and 4(b)). As intended, YQHP and simvastatin impeded the production of Th17 (Figures 4(a) and 4(b)). Levels of CD25^+^Foxp3^+^CD4^+^ Treg cells elevated in mice treated with western diet and HSP65 in feedback to increased Th17 (Figures 4(a) and 4(b)), but the ratio of Th17/Treg was still out of balance, which was reversed by simvastatin and YQHP (Figure 4(c)).

As the surface antigen of CD3 leads to the activation of TCR [32, 33], we next determined the levels in CD4^+^ lymphocytes. Results showed that YQHP, not simvastatin, controlled the percentage of CD3^+^CD4^+^ T cells increased by a high-fat diet, which further extended owing to additional HSP65 assault (Figures 4(d) and 4(e)). From these results, we noticed the regulatory functions of YQHP in Th17 differentiation and TCR activation.

### 3.5. YQHP Regulates Th17 and Treg Differentiation through IL-6/SOCS3-p-STAT3 and IL-2-p-STAT5 Pathway

**Figure 5:**
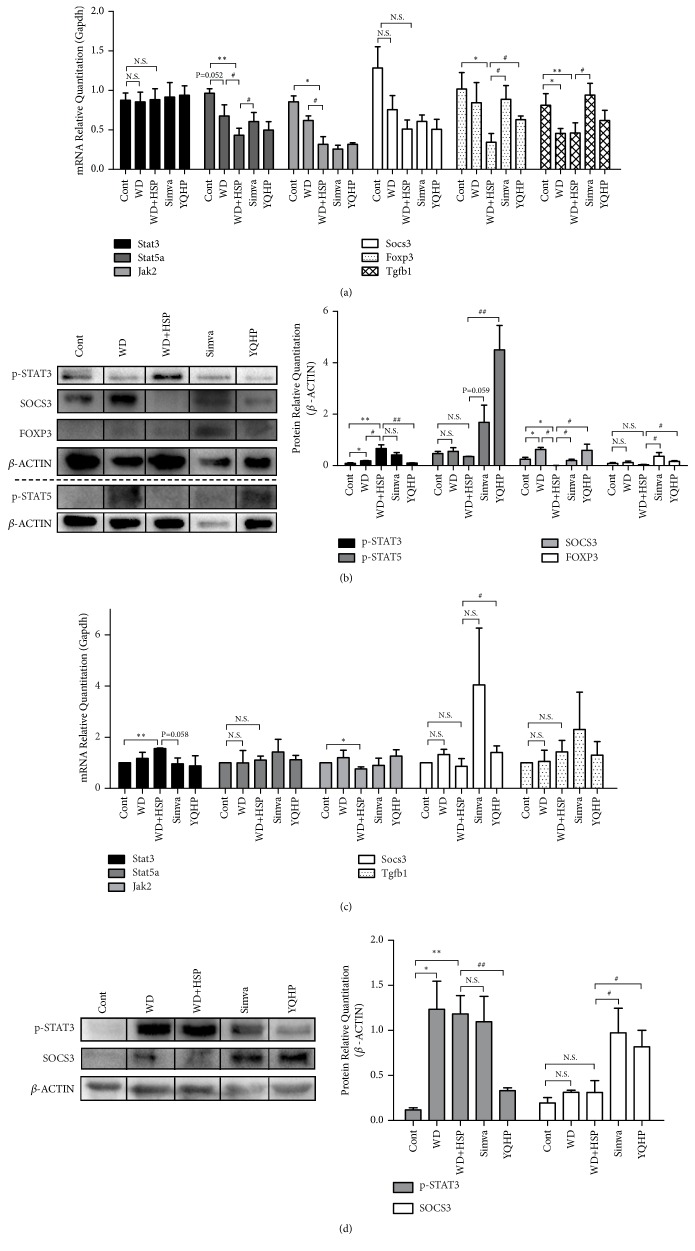
*The effects of YQHP on the mRNA or protein levels of inflammatory factors in spleens or aortas. *(a) The mRNA levels of Stat3, Stat5a, Jak2, Socs3, Foxp3, and Tgfb1 in spleens determined by qPCR (with reference to Gapdh). (b) Western Blot results of spleen p-STAT3, p-STAT5, SOCS3, and FOXP3 protein expression (gray values of target protein/*β*-ACTIN). The top and bottom of the dotted line indicate two gels. (c) The mRNA levels of Stat3, Stat5a, Jak2, Socs3, and Tgfb1 in aortas determined by qPCR (with reference to Gapdh). (d) Western Blot results of spleen p-STAT3 and SOCS3 protein expression (gray values of target protein/*β*-ACTIN). Spleens and aortas from ApoE^−/−^ mice were neatly stripped after 20 weeks. Bars are expressed as mean; error bars: SEM; n value: 3; ^*∗*^P<0.05, ^*∗∗*^P<0.01 versus the control group; ^#^P<0.05, ^##^P<0.01 versus the WD+HSP group; N.S.: not significant.

Since YQHP regulated IL-6 and IL-2 levels in serum (Figures 3(a) and 3(d)) as well as modified numbers of Th17 and Treg cells (Figures 4(a) and 4(b)), we investigated the factors participating in the differentiation of the two kinds of cells in spleens and aortas. As the differentiation from naïve CD4^+^ T cells towards Th17 is attributed to IL-6-STAT3 crosstalk [44], we determined the expression of STAT3 mRNA and its phosphorylation at the protein level. No change of STAT3 was detected at the transcriptional level in spleens or aortas of drug groups, but the double attack with western diet and HSP65 raised its mRNA level only in aortas (Figures 5(a) and 5(c)). In terms of phosphorylation, western diet raised the level of phosphorylated STAT3 (p-STAT3) both in spleens and aortas, and a further advance promoted by HSP65 that arose in spleens (Figures 5(b) and 5(d)) was synergistic with the changes in Th17 cells (Figures 4(a) and 4(b)) in peripheral blood. Oppositely, YQHP ideally restored its phosphorylation levels both in spleens and aortas (Figures 5(b) and 5(d)), which represents suppressed responses to IL-6.

Because SOCS3 indirectly blocks phosphorylation of STAT1 and STAT3 involving IL-10-JAK pathway [59], and the level of IL-10 in serum was already altered (Figure 3(f)), we next studied the levels of JAK2 and SOCS3. Depressed JAK2 mRNA was discovered in both spleens and aortas in western diet plus HSP65 group, which could not be altered by YQHP (Figures 5(a) and 5(c)) and showed no change at translation level in each group (data not shown). In terms of SOCS3 at the transcriptional level, we only observed the change in YQHP-treated aortas compared to that in the western diet plus HSP65 group, but no influence appeared under the action of HSP65 or western diet (Figures 5(a) and 5(c)). Nevertheless, the expression of SOCS protein was almost not determined in spleens treated with western diet and HSP65 (Figure 5(b)), in contrast to elevated levels both in spleens and aortas under the intervention of simvastatin or YQHP (Figures 5(b) and 5(d)). These results showed that the avianized phosphorylation of STAT3 conducted by YQHP may be carried out through enhanced SOCS3 and inhibited IL-6 under high-fat and inflammatory circumstances.

On the other hand, the amount of Treg cells increased under modeling, while only faint variety was observed in drug groups (Figures 4(a) and 4(b)). Even so, the level of phosphorylated STAT5 (p-STAT5) in spleens was elevated with YQHP treatment (Figure 5(b)), which supervises the differentiation towards Treg [37, 38] in response to the enhanced IL-2 signal (Figure 3(d)). As a downstream signal of IL-2-p-STAT5 signaling [37], the expression of Foxp3 mRNA or protein in spleens was upregulated via YQHP or simvastatin treatment, in contrast with the declined transcriptional level in the HSP65 group (Figures 5(a) and 5(b)). However, no change of p-STAT5 was observed in aortas (data not shown).

### 3.6. YQHP Modifies Costimulatory Effects in Diseased Aortas

**Figure 6:**
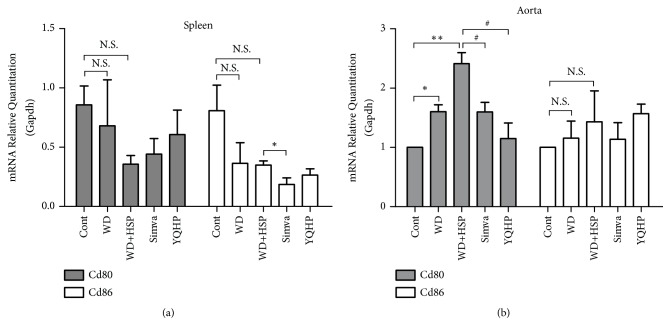
*The effects of YQHP on the mRNA levels of costimulatory molecules in spleens or aortas. *(a) The mRNA levels of Cd80 and Cd86 in spleens determined by qPCR (with reference to Gapdh). (b) The mRNA levels of Cd80 and Cd86 in aortas determined by qPCR (with reference to Gapdh). Spleens and aortas from ApoE^−/−^ mice were neatly stripped after 20 weeks. Bars are expressed as mean; error bars: SEM; n value: 5; ^*∗*^P<0.05, ^*∗∗*^P<0.01 versus the control group; ^#^P<0.05 versus the WD+HSP group; N.S.: not significant.

In consideration of the variety of GM-CSF in serum (Figure 3(g)), the inducer of APCs, as well as the altered levels of CD3^+^CD4^+^ TCR-activated T cells, we inspected the transcriptional levels of costimulators. Reduced level of CD86 mRNA was confirmed in spleens treated with simvastatin compared to that in western diet plus HSP65 group, while no change was observed during treatment of western diet or HSP65 (Figure 6(a)). But the level of CD80 mRNA in aortas was obviously upregulated in hyperlipidemia milieu and was further raised by HSP65, which can be reserved by simvastatin or YQHP (Figure 6(b)), indicating the weakened T cell stimulating capability of APCs linked to restrained T cell response in AS lesion areas as a result of drug intervention.

## 4. Discussion

Although AS can be induced and promoted by a high-fat diet, a large amount of evidence stated clearly the accompaniment of inflammation in the pathogenesis and progress of AS, which was firstly proposed by Ross [60]. Here, HSP65 is a suitable agent to study the relationship between inflammation and AS. As a high-risk autoantigen in AS, it stimulates response from self-reactive T cells [47] and promotes the production of anti-HSP65 antibodies, which is related to CD14 signaling and p38 mitogen-activated protein kinase in the interaction of vascular endothelial cells and macrophages resembling the behavior of lipopolysaccharide (LPS) [61], which lead to inflammatory response and endothelial burden and finally promote the progression of AS. Therefore, apart from WD group, we added WD+HSP group as another model group to investigate the impact of HSP65-mediated inflammation on AS progression in the context of a high-fat diet, compared with high-fat diet alone. As a result, on the basis of high-fat diet, the development of AS lesions in ApoE^−/−^ mice can be accelerated successfully through HSP65 injection. The results indicated that the onset of AS in ApoE^−/−^ mice is bound up with both high-fat diet and infective inflammation.

Although simvastatin is a hypolipidemic agent widely used in the clinic, it also shows exact anti-inflammatory effects in AS [55, 56], autoimmune disorder [62], and other inflammatory diseases [63]. In the current research, it was found that the curative effects of YQHP on AS, i.e., lipid-regulating and inflammation-controlling functions, are identical to simvastatin, which may be attributed to multiple active ingredients in YQHP. For instance, rhizoma polygonati polysaccharide from* Polygonatum sibiricum* [64, 65] and resveratrol [66, 67] from* Reynoutria japonica* improved serum lipids in hyperlipidemia-mediated AS mice and attenuated IL-6-p-STAT3 signaling. Analogous effects were confirmed during treatment of curcumin from* Curcuma longa* [68, 69], which also controls aberrant T cell response and Th1/Th2 balance [70]. However, the application of simvastatin can lead to nonnegligible hepatotoxicity [52, 53], which will pose a burden on patients suffering from AS. Although the hepatotoxicity of* Reynoutria multiflora* has been confirmed [71, 72], it was remarkably reduced and was lower than simvastatin after compatibility with other Chinese medicines in YQHP. Therefore, compared to simvastatin, YQHP showed better treatment effects on atherosclerosis development and lower hepatotoxicity.

Multiple researches have testified the importance of HDL-C in the control of AS [73, 74]. The impact of HSP65 on blood lipids focused on HDL-C rather than TC, TG or LDL-C, which resembles previous studies [75]. The lipid-managing function of YQHP was similar to simvastatin. The hyperlipidemia was not controlled at 10th week, but decreased levels of TC, TG, and LDL-C and elevated HDL-C were observed at 20th weeks of YQHP or simvastatin intervention, hinting the significance of long-term treatment.

Additionally, it has been validated that HDL-C could modulate immune cells in AS via the interference of intracellular metabolism of cholesterol [76, 77]. The changes of immune cells caused by HSP65 via downregulation of HDL-C may be related to the aggravation of AS lesions. Therefore, restoration of HDL-C levels through YQHP might play a part in the regulation of immune cells during the progression of AS. Moreover, in terms of inflammation regulation, inflammatory factors such as IL-6 and IL-22, and Th17 differentiation were induced by western diet, while HSP65 weakened the production of protective cytokines including IL-2 and TGF-*β* and further aggrandized the proliferation of Th17, which brought about pathological shift of the balance of Th17/Treg cells, indicating the dominated pathological state of Th17 in AS. YQHP showed different anti-inflammatory effects than simvastatin, particularly on the powerful suppression of IL-6 and STAT3 phosphorylation, which responded to IL-6 and led to a declined ratio of Th17/Treg. The augment of SOCS3 and downregulation of IL-10 may be interrelated with attenuated IL-6/p-STAT3 signaling and Th17/Treg ratio as well. However, the aggrandizement of protective mechanisms involving IL-2 production, STAT5 phosphorylation, and the polarization of Tregs were faintish.

It is well-known that antigen presentation by APCs leads to activation of local T cell and secretion of inflammatory cytokines, which promote the progress of AS [78, 79]. GM-CSF, which is a stimulating factor of APCs [57], increased in hyperlipidemia environment. Incremental expression of first signal responsive CD3 in T cells and enhanced costimulatory molecule CD80 in aortas were also recognized in high-fat diet fed mice with or without HSP65 treatment, which is related to the T cell activation effects of APCs. Nevertheless, YQHP could reverse the increased production of GM-CSF and the elevated levels of CD3^+^ CD4^+^ T cells or CD80 mRNA, hinting potential effects on stimulation and response of APCs.

**Figure 7:**
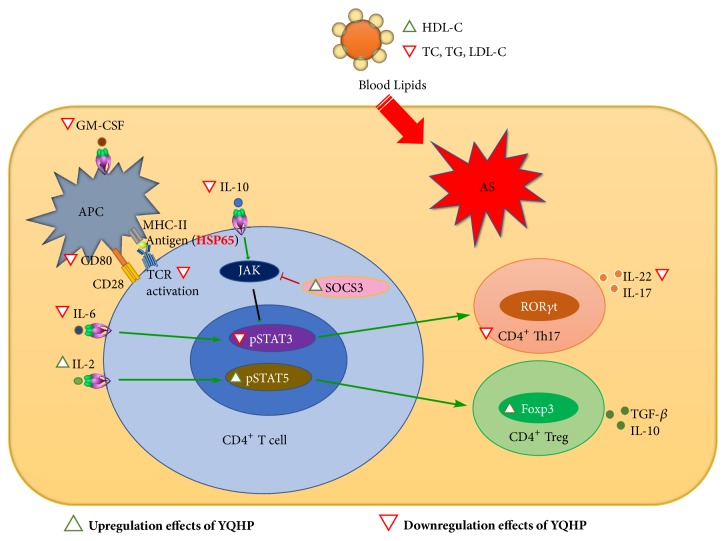
*Summary diagram characterizing the effects of YQHP on AS.* In hyperlipidemia-mediated and autoantigen-aggravated AS, apart from the accommodation of blood lipids, YQHP influenced the production of APC-inducing GM-CSF, transcription of costimulators, and expression of CD3 in CD4^+^ T cells. And the regulation of IL-6/IL-10/SOCS3-pSTAT3 and IL-2-pSTAT5 signaling may participate in the adjustment of Th17/Treg balance, which probably led to the variety of levels of Foxp3 and IL-22. These effects exceedingly potentially promoted the improvement of AS.

In summary, high-fat diet and subcutaneous infection of HSP65 can obviously induce inflammation-based AS in ApoE^−/−^ mice, while YQHP exerts excellent effects on the complicated atherogenic condition. Proper management of blood lipids, appropriate control of T cell activation, powerful inhibition of IL-6-p-STAT3 (Figure 7) and low hepatotoxicity reveal the superiority of the principle of formulating Yangyin Qingre Huoxue recipe during traditional treatment of AS, which is in demand of long-term intervention.

## Figures and Tables

**Table 1:** Plant names and therapeutic effects of Chinese medicine used in YQHP.

Plant names	Chinese / English names	Therapeutic effects	References
*Reynoutria multiflora* (Thunb.) Moldenke	Heshouwu / Tuber Fleeceflower Root	Lipid-regulation; anti-inflammation; anti-oxidation; anti-tumor; purgation; diuresis; cholagogue; neuroprotection	[17, 18]
*Polygonatum sibiricum* F. Delaroche	Huangjing / Manyflower Solomonseal Rhizome	Lipid-regulation; glucose reduction; senility prevention	[19, 20]
*Reynoutria japonica *Houtt.	Huzhang / Giant Knotweed Rhizome	Anti-inflammation (including hepatitis); anti-tumor; anti-diarrhea; diuresis; detoxification	[21, 22]
*Stemmacantha uniflora* (L.) Dittrich	Loulu / Uniflower Swisscentaury Root	Dissipation of indurated mass; detoxification	[23]
*Curcuma longa* L.	Jianghuang / Turmeric	Lipid-regulation; anti-inflammation (including atherosclerosis and arthritis); anti-oxidation; wound healing; neuroprotection; senility prevention; glucose reduction; anti-tumor; anti-infection	[24–26]
*Carthamus tinctorius* L.	Honghua / Safflower	Cardiovascular and cerebrovascular improvement (including coronary heart diseases, angina pectoris, hypertension and stroke); lipid-regulation; anti-inflammation (including angiitis); anti-gynecopathy	[27–29]

**Table 2:** Animal modeling and drug delivery.

Group (n=10/group)	Treatment	Drug delivery (i.g.)
Control (Ctrl)	Normal feed	Normal saline (0.001ml/kg)
Western diet (WD)	High-fat feed	Normal saline (0.001ml/kg)
Western diet plus HSP65 (WD+HSP)	High-fat feed and mHSP65 (s.c.)	Normal saline (0.001ml/kg)
Simvastatin (Simva)	High-fat feed and mHSP65 (s.c.)	Simvastatin (3.3 mg/kg)
Yangyin Qingre Huoxue Prescription (YQHP)	High-fat feed and mHSP65 (s.c.)	YQHP (18 g/kg)

## Data Availability

The data used to support the findings of this study are available from the corresponding author upon request.

## Supplementary Materials

Supplementary Materials Figure S1: HPLC chromatographic peaks of quality inspection components in YQHP. Figure S2: comparison of hepatotoxicity between simvastatin and YQHP in hyperlipidemia. Table S1: the content of inspection components in YQHP measured by HPLC.

## References

[1] Taleb S., Tedgui A., Mallat Z. (2015). IL-17 and Th17 cells in atherosclerosis: subtle and contextual roles. *Arteriosclerosis, Thrombosis, and Vascular Biology*.

[2] Melzer S., Ankri R., Fixler D., Tarnok A. (2015). Nanoparticle uptake by macrophages in vulnerable plaques for atherosclerosis diagnosis. *Journal of Biophotonics*.

[3] Stefano R., Massimiliano C., Marina C. (2015). A score including ADAM17 substrates correlates to recurring cardiovascular event in subjects with atherosclerosis. *Atherosclerosis*.

[4] Legein B., Temmerman L., Biessen E. A. L., Lutgens E. (2013). Inflammation and immune system interactions in atherosclerosis. *Cellular and Molecular Life Sciences*.

[5] Kusters P. J. H., Lutgens E. (2015). Cytokines and immune responses in murine atherosclerosis. *Methods in Molecular Biology*.

[6] Jiang W., Tang S. (2011). Application of stagnant heat pathogenesis theory in the differentiation and treatment of cardiovascular diseases. *Lishizhen Medicine and Materia Medica Research*.

[7] Wang Q., Ren X. J., Yao S. L., Wu H. D. (2010). Clinical observation on the endocrinal and immune functions in subjects with yin-deficiency constitution. *Chinese Journal of Integrative Medicine*.

[8] Sun Y. (2011). Discussion on relationship between arteriosclerosis and the theory of heat and toxin. *Liaoning University of Traditional Chinese Medicine*.

[9] China Pharmacopoeia Committee (2015). *Pharmacopoeia of the People’s Republic of China*.

[10] Sun Y. (2006). Advancement in research of dealing with artherosclerosis with Chinese medicine. *Jiangsu Journal of Traditional Chinese Medicine*.

[11] Sun Y. (2007). Discussion on inflammatory pathogeny of angina pectoris and it's TCM treatment. *Jiangsu Journal of Traditional Chinese Medicine*.

[12] Ning C. (2017). Clinical study on treatment of 30 cases of hyperlipemia of liver and kidney yin deficiency with "Luhuang granule". *Jiangsu Journal of Traditional Chinese Medicine*.

[13] Sun Y. (2008). Clinical study on Chinese medicine intervention for carotid atherosclerosis and inflammatory factors in coronary heart disease. *Liaoning Journal of Traditional Chinese Medicine*.

[14] Wu W., Wang Z. (2018). The experience of treating coronary heart disease by professor TANG Shuhua. *Zhejiang Chinese Medical University (ZCMU)*.

[15] Ma Y., Qiu R., Yuan D., Long J. (2018). Effects of YHF on expression of Treg in apolipoprotein E knockout mice. *Traditional Chinese Drug Research & Clinical Pharmacology*.

[16] Zhang G., Xie D., Yang Y. (2015). Effect of Luhuang granule on levels of hypersensitivity C reactive protein and Lipids in rabbits with atherosclerosis. *Jiangxi Medical Journal*.

[17] Zheng C.-J., Zhao S.-J., Shao L. (2014). Species identification of Chinese medicinal plant Fallopia multiflora (Thunb.) Haraldson by suppression subtraction hybridization. *Journal of Natural Medicines*.

[18] Zhao W., Xia W., Li J., Sheng S., Lei L., Zhao S. (2014). Transcriptome profiling and digital gene expression analysis of* Fallopia multiflora* to discover putative genes involved in the biosynthesis of 2,3,5,4^'^-tetrahydroxy stilbene-2-O-*β*-d-glucoside. *Gene*.

[19] Zhang H., Cao Y., Chen L. (2015). A polysaccharide from Polygonatum sibiricum attenuates amyloid-*β*-induced neurotoxicity in PC12 cells. *Carbohydrate Polymers*.

[20] Ko J. H., Kwon H. S., Yoon J. M. (2015). Effects of Polygonatum sibiricum rhizome ethanol extract in high-fat diet-fed mice. *Pharmaceutical Biology*.

[21] Chen H., Tuck T., Ji X. (2013). Quality assessment of Japanese knotweed (Fallopia japonica) grown on Prince Edward Island as a source of resveratrol. *Journal of Agricultural and Food Chemistry*.

[22] Hwangbo K., Zheng M. S., Kim Y.-J. (2012). Inhibition of DNA topoisomerases i and II of compounds from Reynoutria japonica. *Archives of Pharmacal Research*.

[23] Xu H., Li M., Wang C. (2018). Evaluation on clinical efficacy of Fuzheng Jiedu Huayu Decoction combined with antibiotics in the treatment of pneumonia in the elderly – A multi-center, double-blind, parallel, randomized controlled trial. *Complementary Therapies in Medicine*.

[24] Han J.-M., Lee J.-S., Kim H.-G. (2015). Synergistic effects of Artemisia iwayomogi and Curcuma longa radix on high-fat diet-induced hyperlipidemia in a mouse model. *Journal of Ethnopharmacology*.

[25] Joshi D., Mittal D. K., Shukla S., Srivastav S. K., Dixit V. A. (2017). Curcuma longa Linn. extract and curcumin protect CYP 2E1 enzymatic activity against mercuric chloride-induced hepatotoxicity and oxidative stress: A protective approach. *Experimental and Toxicologic Pathology*.

[26] Araújo L. A., Araújo R. G., Gomes F. O. (2016). Physicochemical/photophysical characterization and angiogenic properties of Curcuma longa essential oil. *Anais da Academia Brasileira de Ciências*.

[27] Junlatat J., Sripanidkulchai B. (2014). Hair growth-promoting effect of Carthamus tinctorius floret extract. *Phytotherapy Research*.

[28] Chen J., Deng J., Zhang Y. (2014). Lipid-lowering effects of Danhong injection on hyperlipidemia rats. *Journal of Ethnopharmacology*.

[29] Wang Y., Chen P., Tang C., Li Y., Zhang H. (2014). Antinociceptive and anti-inflammatory activities of extract and two isolated flavonoids of *Carthamus tinctorius* L. *Journal of Ethnopharmacology*.

[30] Cochain C., Chaudhari S. M., Koch M. (2014). Programmed Cell Death-1 Deficiency Exacerbates T Cell Activation and Atherogenesis despite Expansion of Regulatory T Cells in Atherosclerosis-Prone Mice. *PLoS ONE*.

[31] Ketelhuth D. F., Gistera A., Johansson D. K., Hansson G. K. (2013). T cell-based therapies for atherosclerosis. *Current Pharmaceutical Design*.

[32] Villadangos J. A., Vega-Ramos J., Roquilly A., Asehnoune K. (2014). Modulation of dendritic cell antigen presentation by pathogens, tissue damage and secondary inflammatory signals. *Current Opinion in Pharmacology*.

[33] Shi X., Bi Y., Yang W. (2013). Ca^2+^ regulates T-cell receptor activation by modulating the charge property of lipids. *Nature*.

[34] Liang Y., Cucchetti M., Roncagalli R. (2013). The lymphoid lineage-specific actin-uncapping protein Rltpr is essential for costimulation via CD28 and the development of regulatory T cells. *Nature Immunology*.

[35] Potekhina A. V., Pylaeva E., Provatorov S. (2015). Treg/Th17 balance in stable CAD patients with different stages of coronary atherosclerosis. *Atherosclerosis*.

[36] Zhu M., Mo H., Li D., Luo X., Zhang L. (2013). Th17/Treg imbalance induced by increased incidence of atherosclerosis in patients with systemic lupus erythematosus (SLE). *Clinical Rheumatology*.

[37] Bailey-Bucktrout S. L., Martinez-Llordella M., Zhou X. (2013). Self-antigen-driven activation induces instability of regulatory T cells during an inflammatory autoimmune response. *Immunity*.

[38] Huss D. J., Mehta D. S., Sharma A. (2014). In Vivo Maintenance of Human Regulatory T Cells during CD25 Blockade. *The Journal of Immunology*.

[39] Dardalhon V., Awasthi A., Kwon H. (2008). IL-4 inhibits TGF-*β*-induced Foxp3^+^ T cells and, together with TGF-*β*, generates IL-9^+^ IL-10^+^ Foxp3^−^ effector T cells. *Nature Immunology*.

[40] Bommireddy R. (2003). TGF beta 1 inhibits Ca2+-calcineurin-mediated activation in thymocytes. *The Journal of Immunology*.

[41] Li M. O., Wan Y. Y., Flavell R. A. (2007). T cell-produced transforming growth. *Immunity*.

[42] Subramanian M., Thorp E., Hansson G. K., Tabas I. (2013). Treg-mediated suppression of atherosclerosis requires MYD88 signaling in DCs. *The Journal of Clinical Investigation*.

[43] Li F., Ji L., Wang W. (2015). Insufficient secretion of IL-10 by Tregs compromised its control on over-activated CD4+ T effector cells in newly diagnosed adult immune thrombocytopenia patients. *Immunologic Research*.

[44] Wang Y., Xing F., Ye S. (2015). Jagged-1 signaling suppresses the IL-6 and TGF-*β* treatment-induced Th17 cell differentiation via the reduction of ROR*γ*t/IL-17A/IL-17F/IL-23a/IL-12rb1. *Scientific Reports*.

[45] Ramesh R., Kozhaya L., McKevitt K. (2014). Pro-inflammatory human Th17 cells selectively express P-glycoprotein and are refractory to glucocorticoids. *The Journal of Experimental Medicine*.

[46] Shah P. K., Chyu K. Y., Dimayuga P. C., Nilsson J. (2014). Vaccine for atherosclerosis. *JACC: Journal of the American College of Cardiology*.

[47] Grundtman C., Kreutmayer S. B., Almanzar G., Wick M. C., Wick G. (2011). Heat shock protein 60 and immune inflammatory responses in atherosclerosis. *Arteriosclerosis, Thrombosis, and Vascular Biology*.

[48] Grundtman C., Wick G. (2011). The autoimmune concept of atherosclerosis. *Current Opinion in Lipidology*.

[49] Xiong Q., Li J., Jin L., Liu J., Li T. (2009). Nasal immunization with heat shock protein 65 attenuates atherosclerosis and reduces serum lipids in cholesterol-fed wild-type rabbits probably through different mechanisms. *Immunology Letters*.

[50] Jun L., Jie L., Dongping Y. (2012). Effects of nasal immunization of multi-target preventive vaccines on atherosclerosis. *Vaccine*.

[51] Osborn E. A., Jaffer F. A. (2013). Imaging atherosclerosis and risk of plaque rupture. *Current Atherosclerosis Reports*.

[52] Björnsson E., Jacobsen E. I., Kalaitzakis E. (2012). Hepatotoxicity associated with statins: reports of idiosyncratic liver injury post-marketing. *Journal of Hepatology*.

[53] Cho Y., Moon P., Lee J. (2013). Integrative analysis of proteomic and transcriptomic data for identification of pathways related to simvastatin-induced hepatotoxicity. *Proteomics*.

[54] Robles-Diaz M., Garcia-Cortes M., Medina-Caliz I. (2015). The value of serum aspartate aminotransferase and gamma-glutamyl transpetidase as biomarkers in hepatotoxicity. *Liver International*.

[55] Yin M., Liu Q., Yu L. (2017). Downregulations of CD36 and Calpain-1, Inflammation, and Atherosclerosis by Simvastatin in Apolipoprotein e Knockout Mice. *Journal of Vascular Research*.

[56] Liu M., Yu Y., Jiang H. (2013). Simvastatin suppresses vascular inflammation and atherosclerosis in ApoE^−/−^ mice by downregulating the HMGB1-RAGE axis. *Acta Pharmacologica Sinica*.

[57] Zhan Y., Xu Y., Lew A. M. (2012). The regulation of the development and function of dendritic cell subsets by GM-CSF: more than a hematopoietic growth factor. *Molecular Immunology*.

[58] Codarri L., Gyülvészii G., Tosevski V. (2011). ROR*γ*3t drives production of the cytokine GM-CSF in helper T cells, which is essential for the effector phase of autoimmune neuroinflammation. *Nature Immunology*.

[59] Neuper T., Ellwanger K., Schwarz H., Kufer T. A., Duschl A., Horejs-Hoeck J. (2017). NOD1 modulates IL-10 signalling in human dendritic cells. *Scientific Reports*.

[60] Ross R. (1999). Atherosclerosis—an inflammatory disease. *The New England Journal of Medicine*.

[61] Milioti N., Bermudez-Fajardo A., Penichet M. L., Oviedo-Orta E. (2008). Antigen-induced immunomodulation in the pathogenesis of atherosclerosis. *Clinical and Developmental Immunology*.

[62] Zhang X., Tao Y., Troiani L., Markovic-Plese S. (2011). Simvastatin inhibits IFN regulatory factor 4 expression and Th17 cell differentiation in CD4+ T cells derived from patients with multiple sclerosis. *The Journal of Immunology*.

[63] Liu J., Suh D., Yang E., Lee S., Park H., Shin Y. S. (2014). Attenuation of airway inflammation by simvastatin and the implications for asthma treatment: is the jury still out?. *Experimental & Molecular Medicine*.

[64] Zhu X., Li Q., Lu F., Wang H., Yan S. (2015). Antiatherosclerotic potential of Rhizoma polygonati polysaccharide in hyperlipidemia-induced atherosclerotic hamsters. *Drug Research*.

[65] Sun L., Wang Y., Song Y., Cheng X. R. (2015). Resveratrol restores the circadian rhythmic disorder of lipid metabolism induced by high-fat diet in mice. *Biochemical and Biophysical Research Communications*.

[66] Liu J., Zhang Q., Chen K. (2012). Small-molecule STAT3 signaling pathway modulators from polygonum cuspidatum. *Planta Medica*.

[67] Li Y., Zhu W., Li J., Liu M., Wei M. (2013). Resveratrol suppresses the STAT3 signaling pathway and inhibits proliferation of high glucose-exposed HepG2 cells partly through SIRT1. *Oncology Reports*.

[68] Sahebkar A. (2014). A systematic review and meta-analysis of randomized controlled trials investigating the effects of curcumin on blood lipid levels. *Clinical Nutrition*.

[69] Duan W., Yang Y., Yan J. (2012). The effects of curcumin post-treatment against myocardial ischemia and reperfusion by activation of the JAK2/STAT3 signaling pathway. *Basic Research in Cardiology*.

[70] Zheng M., Zhang Q., Joe Y. (2013). Curcumin induces apoptotic cell death of activated human CD4+ T cells via increasing endoplasmic reticulum stress and mitochondrial dysfunction. *International Immunopharmacology*.

[71] Lin L., Lin H., Zhang M. (2015). A novel method to analyze hepatotoxic components in Polygonum multiflorum using ultra-performance liquid chromatography-quadrupole time-of-flight mass spectrometry. *Journal of Hazardous Materials*.

[72] Lin L., Li H., Lin H. (2018). Application of iTRAQ-Based Quantitative Proteomics Approach to Identify Deregulated Proteins Associated with Liver Toxicity Induced by Polygonum Multiflorum in Rats. *Cellular Physiology and Biochemistry*.

[73] Calabresi L., Gomaraschi M., Simonelli S., Bernini F., Franceschini G. (2015). HDL and atherosclerosis: Insights from inherited HDL disorders. *Biochimica et Biophysica Acta (BBA) - Molecular and Cell Biology of Lipids*.

[74] Mei X., Atkinson D. (2015). Lipid-free apolipoprotein A-I structure: insights into HDL formation and atherosclerosis development. *Archives of Medical Research*.

[75] Sun H., Shen J., Liu T. (2014). Heat shock protein 65 promotes atherosclerosis through impairing the properties of high density lipoprotein. *Atherosclerosis*.

[76] Catapano A. L., Pirillo A., Bonacina F., Norata G. D. (2014). HDL in innate and adaptive immunity. *Cardiovascular Research*.

[77] Ye D., Lammers B., Zhao Y., Meurs I. (2011). ATP-binding cassette transporters A1 and G1, HDL metabolism, cholesterol efflux, and inflammation: important targets for the treatment of atherosclerosis. *Current Drug Targets*.

[78] Koltsova E. K., Garcia Z., Chodaczek G. (2012). Dynamic T cell-APC interactions sustain chronic inflammation in atherosclerosis. *The Journal of Clinical Investigation*.

[79] Taghavie-Moghadam P. L., Butcher M. J., Galkina E. V. (2014). The dynamic lives of macrophage and dendritic cell subsets in atherosclerosis. *Annals of the New York Academy of Sciences*.

