# Supplementary material for: Yangyin Qingre Huoxue Method in Traditional Chinese Medicine Ameliorates Atherosclerosis in ApoE−/− Mice Suffering from High-Fat Diet and HSP65 Aggression
Source: Evid Based Complement Alternat Med. 2019 Jan 1;2019:2531979. doi: 10.1155/2019/2531979 (PMC6332951; doi:10.1155/2019/2531979)
Supplement: Supplementary Materials — Figure S1: HPLC chromatographic peaks of quality inspection components in YQHP. Figure S2: comparison of hepatotoxicity between simvastatin and YQHP in hyperlipidemia. Table S1: the content of inspection components in YQHP measured by HPLC. [file 2531979.f1.docx]

Yangyin Qingre Huoxue Method in Traditional Chinese Medicine Ameliorates Atherosclerosis in ApoE^-/-^ Mice Suffered from High-fat Diet and HSP65 Aggression

- Supplementary materials -

Runze Qiu^a,#^, Jun Long^a,#^, Liyu Zhou^a^, Yuanjing Ma^a^, Lingang Zhao^b^, Fumin Liu^b^, Dongping Yuan^a,*^

^a^ School of Pharmacy, Jiangsu Key Laboratory for Pharmacology and Safety Evaluation of Chinese Materia Medica, Nanjing University of Chinese Medicine, Xianlin Dadao 138, Nanjing, 210023, P.R. China

^b^ Jiangsu Province Hospital of TCM, Affiliated Hospital of Nanjing University of Traditional Chinese Medicine, Hanzhong Road 155, Nanjing, 210029, P.R. China

^#^ These authors contributed equally to this work.

^*^Corresponding Author:

Dongping Yuan, Ph.D

School of Pharmacy

Nanjing University of Chinese Medicine

Xianlin Dadao 138

Nanjing, 210023

Jiangsu, P.R. China

Fax: 86-025-85811153

E-mail: [annieyuan99@163.com](mailto:annieyuan99@163.com)

Email addresses of other authors:

Runze Qiu (first author): [watato@liblu.me](mailto:watato@liblu.me)

Jun Long (first author): [long_ydp@aliyun.com](mailto:long_ydp@aliyun.com)

Liyu Zhou: [liyuzhou210@sina.com](mailto:liyuzhou210@sina.com)

Yuanjing Ma: [mayuanjing93@163.com](mailto:mayuanjing93@163.com)

Lingang Zhao: [zhaolingang001@sina.com](mailto:zhaolingang001@sina.com)

Fumin Liu: [doctor.liufuming@outlook.com](mailto:doctor.liufuming@outlook.com)


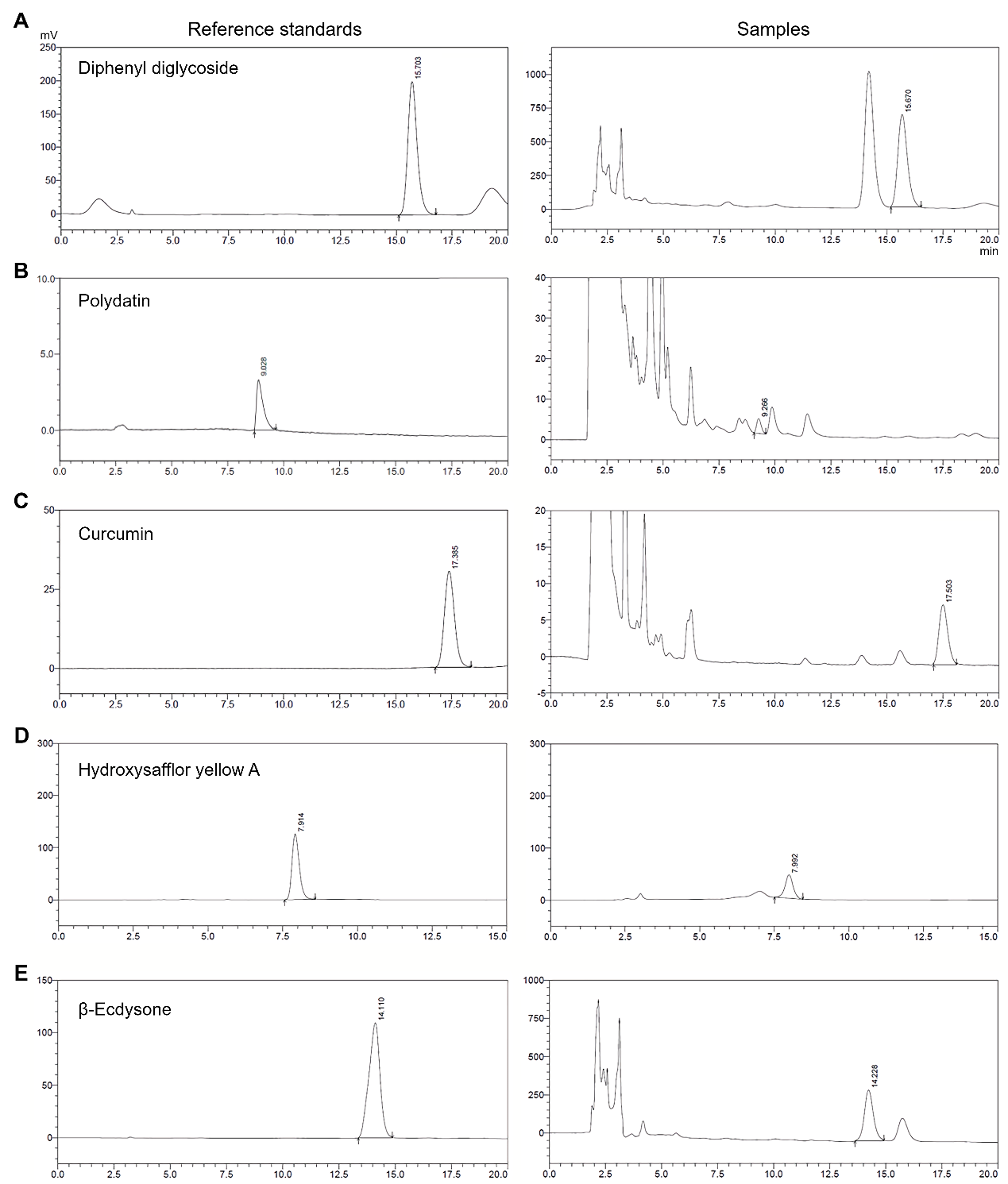


**Figure S1. HPLC chromatographic peaks of quality inspection components in YQHP.** (**A**) The peak of Diphenyl diglycoside in YQHP at 320nm (mobile phase: 17% acetonitrile and 83% water). (**B**) The peak of Polydatin in YQHP at 300nm (mobile phase: 40% acetonitrile and 0.2% phosphoric acid solution). (**C**) The peak of Curcumin in YQHP at 425nm (mobile phase: 48% acetonitrile and 0.2% glacial acetic acid solution). (**D**) The peak of Hydroxysafflor yellow A in YQHP at 403nm (mobile phase: 26% methanol, 2% acetonitrile and 0.2% phosphoric acid solution). (**E**) The peak of β-Ecdysone in YQHP at 247nm (mobile phase: 17% acetonitrile and 83% water).

**
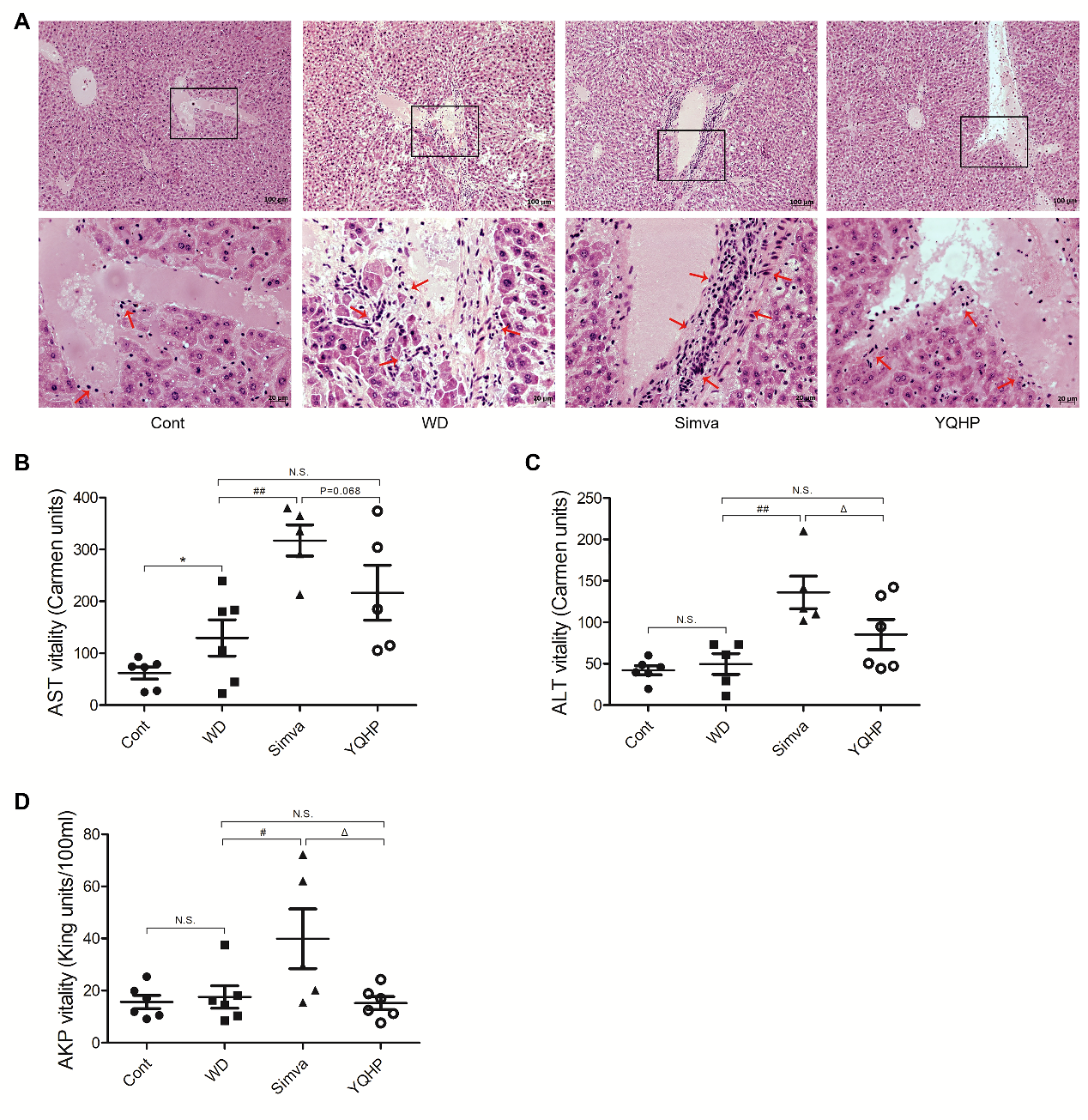
Figure S2. Comparison of hepatotoxicity between Simvastatin and YQHP in hyperlipidemia.** (**A**) The photos of livers of ApoE^-/-^ mice stained by H&E (magnification: × 100 and × 400). (**B**) The levels of AST in serum of ApoE^-/-^ mice. (**C**) The levels of ALT in serum of ApoE^-/-^ mice. (**D**) The levels of AKP in serum of ApoE^-/-^ mice. The dose of simvastatin was the minimum clinical equivalent dosage and YQHP was on the common dose. Livers and serums were collected after 1 months of western diet and drug administration. Black frames in 100 times magnification photos represent photographical areas of 400 times magnification photos and red arrows point to monocyte infiltration areas. The vitality of enzymes was measured by microplate kits. Each circle from scatter dot plots represents the data from one mouse and lines are expressed as mean; error bars: SEM; ^*^P<0.05 versus the control group; ^#^P<0.05, ^##^P<0.01 versus the WD+HSP group (A and B); ^Δ^P<0.05 versus the Simva group.

**Table S1.** The content of inspection components in YQHP measured by HPLC.

| Inspection component | Content in YQHP (%) |
| --- | --- |
| Diphenyl diglycoside | 0.9979 |
| Polydatin | 0.2566 |
| Curcumin | 1.5101 |
| Hydroxysafflor yellow A | 1.5148 |
| β-Ecdysone | 0.1008 |
